# FGFR2/STAT3 Signaling Pathway Involves in the Development of MMTV-Related Spontaneous Breast Cancer in TA2 Mice

**DOI:** 10.3389/fonc.2020.00652

**Published:** 2020-05-05

**Authors:** Jiaxing Du, Qi Zhao, Kai Liu, Zugui Li, Fangmei Fu, Kexin Zhang, Hao Zhang, Minying Zheng, Yongjie Zhao, Shiwu Zhang

**Affiliations:** ^1^Graduate School, Tianjin University of Traditional Chinese Medicine, Tianjin, China; ^2^Department of Pathology, Tianjin Union Medical Center, Tianjin, China; ^3^Graduate School, Tianjin Medical University, Tianjin, China; ^4^Nankai University School of Medicine, Nankai University, Tianjin, China; ^5^Departments of General Surgery, Tianjin Union Medical Center, Tianjin, China

**Keywords:** tientsin albino 2, FGFR2/STAT3, triple-negative breast cancer, spontaneous breast cancer, MMTV

## Abstract

The Tientsin Albino 2 (TA2) mouse has a high incidence of spontaneous breast cancer (SBC) in the absence of external inducers or carcinogens. The initiation of SBC is related to mouse mammary tumor virus (MMTV) infection and pregnancy. Pathologic analysis showed that breast cancer cells in TA2 mice are triple negative. Our previous study confirmed that fibroblast growth factor receptor 2 (FGFR2) expression increased in SBC tissue compared to that in their corresponding normal breast tissues of TA2 mice. The present study focused on the function of the FGFR2/STAT3 signaling pathway in the initiation of SBC. In this study, the expression of FGF3, FGFR2, STAT3, p-STAT3^Tyr705^, and p-STAT3^Ser727^ was detected in serum and normal mammary gland tissues of TA2 mice with different number of pregnancies and SBC. The proliferation, invasiveness, and migration abilities of MA-891 cells from TA2 SBC were compared before and after cryptotanshinone and Stattic treatment. Transient siRNA transfection was used to detect the invasiveness, and migration abilities to avoid the off-targets effects. Downstream protein expression of STAT3 was also detected in MA-891 cells and TA2 xenografts from MA-891 inoculation. In addition, STAT3 expression was analyzed in 139 cases of human breast cancer including 117 cases of non-triple negative breast cancer (non-TNBC) (group I) and 22 cases of triple-negative breast cancer (TNBC) (group II). Results of our study confirmed that MMTV-LTR amplification, and FGFR2, p-STAT3^Tyr705^, p-STAT3^Ser727^ expression increased with the number of pregnancies in the breast tissue of TA2 mice and were the highest in SBC. Serum FGF3 expression of SBC was higher than it of TA2 mice with different number of pregnancies. After STAT3 was inhibited, the abilities of proliferation, invasiveness, and migration in MA-891 decreased and the expression levels of STAT3, p-STAT3^Ser727^, p-STAT3^Tyr705^, Bcl2, cyclin D1, and c-myc in MA-891 and animal xenografts were also down-regulated. In human breast cancer, STAT3 expression was significantly higher in TNBC than that in non-TNBC. Our results showed that the FGFR2/STAT3 signaling pathway may be related to SBC initiation in TA2 mice. Inhibition of STAT3 can decrease proliferation, invasiveness, and migration in MA-891 cells and the growth of TA2 xenografts.

## Introduction

Breast cancer is the most common malignant tumor and the leading cause of death in women. There were more 2.1 million breast cancer cases in 2018 which accounted for one-quarter of all cancers in women (1). Of the 185 countries included in these statistics, women in 154 countries were most commonly diagnosed with breast cancer (2). Tientsin albino 2 (TA2) mice, a model for spontaneous breast cancer (SBC) established by Tianjin Medical University, have a SBC morbidity rate above 84% (3). Our previous studies showed that the initiation of SBC in TA2 mice was associated with pregnancy status, pregnancy frequency, and mouse mammary tumor virus (MMTV) infection. MMTV is a retrovirus with a long terminal repeats (LTRs). It contains hormone-responsive elements (HRE), transcription enhancer factor-1 (TEF-1) family members, and a superantigen (SAG) coded by open reading frame (ORF) (4, 5). Hormone receptors (progestin, glucocorticoid receptors, and androgen receptors) can bind with the HRE in MMTV promoter to promote the expression of MMTV genes. Our previous studies confirmed that combined exogenous estradiol and progesterone treatment induces breast cancer initiation in TA2 mice without ovaries (4). As a retrovirus, MMTV can integrate its genome into TA2 mouse genome. Insertion of viral DNA within or near an oncogene changes the expression of that gene, leading to the initiation and development of cancer (6, 7). The human genome carries many endogenous retrovirus sequences similar to those of MMTV. Six hundred and sixty MMTV-like env sequences were reported in about 38% of human breast cancer tissues but not in normal breast tissues or other cancers (8). Our previous studies demonstrated that SBC in TA2 mice belongs to triple-negative breast cancer (TNBC), which is negative for estrogen receptor (ER), progesterone receptor (PR), and human epidermal growth factor receptor 2 (HER-2) expression (5, 9, 10). TNBC is more likely to affect younger women and the patients' prognosis is poor (5). Moreover, recent studies have shown that signal transducer and activator of transcription 3 (STAT3) is often over-expressed in TNBC and is closely related to TNBC initiation, progression, metastasis, drug resistance and adverse survival outcomes (11–13).

Our previous study showed that fibroblast growth factor-1 (FGF-1) and fibroblast growth factor receptor 2 (FGFR2) contributed to the initiation of SBC in TA2 mice by regulating the cell cycle and promoting cell proliferation. FGF-1 expression in SBC tumor tissue was higher than that in their corresponding matched normal mammary gland tissues in TA2 mice by using an Affymetrix Mouse 430 2.0 array (6, 10). Recently, through high-throughput screening of MMTV insertion sites in mouse mammary tumors, Klijn et al. confirmed that a series of gene loci including *Wnt, fgf, fgfr*, R-spondin (*Rspo*), and platelet-derived growth factor receptor (*Pdgfr*) are directly involved in MMTV-induced mouse mammary tumors (14). Most of the induced breast tumors are mainly at *wnt1* and *fgf3* sites by MMTV infection in wild-type mice (15, 16). Furthermore, FGFR2 is also a common MMTV insertion site (14). High FGFR expression can activate the expression of STAT3 (17). This activation of STAT3 can regulate the expression of its downstream target genes in TNBC cells to promote cell proliferation, migration, and invasion (18). Phosphorylated STAT3 increases tumor cell proliferation, migration, and invasion by increasing the expression of genes such as b-cell lymphoma 2 (*Bcl2*), cyclin D1, and c-myc (19–21).

This study collected SBC and normal breast tissue, protein, and serum samples from TA2 mice with different numbers of pregnancies and measured the expression of FGF3, FGFR2, STAT3, and phosphorylated STAT3. Moreover, the MA-891 cell line derived from SBC of TA2 mice was used to investigate the molecular mechanisms of STAT3 in the initiation of TA2 SBC. Our results showed that the FGFR2/STAT3 signaling pathway may be related to SBC initiation in TA2 mice. Inhibition of STAT3 decreased proliferation, invasiveness, and migration in MA-891 and TA2 xenografts.

## Materials and Methods

### TA2 Mice With SBC

The mice were purchased from Tianjin Medical University and divided into five groups containing five mice each group according to the number of pregnancies, including the non-pregnancy, two pregnancies, four pregnancies, six pregnancies and SBC, which were marked by 0, 2, 4, 6, and SBC, respectively. The mice were raised for more than 14 months and sacrificed according to the experimental requirements. When the number of pregnancies of the mice is more than 6, some mice developed into SBC. The breast cancer tissues, mammary glands, and whole blood from the different groups were collected. The Institutional Animal Care and Use Committee of Tianjin Union Medicine Center approved the animal experimentation protocols and all animal experiments were performed according to the Guidelines for the Care and Use of Laboratory Animals established by the Chinese Council on Animal Care.

### Human Breast Cancer Samples

Paraffin-embedded of human breast cancer samples (*n* = 139) were obtained from Tianjin Union Medical Center (Tianjin, China). The patients had been diagnosed with breast cancer and had not received medical treatment for breast cancer before surgical resection. These 139 cases of human breast cancer were divided into non-TNBC (117 cases, group I) and TNBC (22 cases, group II) groups according to clinicopathological results. The utilization of these tumor samples was permitted by the tissue bank of the Tianjin Union Medical Center and patient information was kept strictly confidential.

### MA-891 Cell Line With Cryptotanshinone (CTS) and Stattic Treatment

The MA-891 cell line was obtained from KeyGEN BioTECH, Inc. (NanJing, China) and maintained in RPMI 1640 medium (Gibco, USA) containing 10% heat-inactivated fetal bovine serum (FBS) (ExCell Biology, USA), penicillin (100 U/mL), and streptomycin (100 μg/mL) in a 5% CO_2_ incubator at 37°C. CTS (Solarbio, China) and Stattic (Selleck Chemicals, USA) were used to treat the cells. Stattic (Selleck Chemicals, USA) and CTS (Solarbio, China) were dissolved in DMSO for different concentration.

### Transient siRNA Transfection

The siRNA sequences targeted to the mouse STAT3 were synthesized by Shanghai Gene-pharma including three siRNA interference sequences, one positive control sequence (GAPDH), one negative control (NC) sequence (sequences of siRNAs have been listed in Supplementary Table 1). Three STAT3 transfection sequences including 2315, 1415, 1107 were used to inhibit the expression of STAT3. When the cells were 60–70% confluent in six-well plates (2 × 10^5^ cells/well), Lipofectamine 2000 (Invitrogen, Carlsbad, CA, USA) and 1× Opti-MEM (Gibco, USA) were used to dilute the STAT3 negative control siRNA or STAT3 siRNA following the manufacturer's protocol, and the mixture was added to the cells. The cells were harvested for 48 h after transfection to examine the effect of targeted protein knockdown with western blots.

### Wound-Healing Assay

Wound-healing assays were used to evaluate the migration abilities of control cells compared to the cells treated with CTS and Stattic. Detailed information was provided in the Supplementary Materials and Methods.

### Cell Viability Cell Counting Kit-8 (CCK8) Assay

MA-891 cells were seeded in 96-well plates at 2,000 cells per well and incubated for 12 h. These cells were divided into groups and every group was repeated in triplicate. Detailed information was provided in the Supplementary Materials and Methods.

### Cell Migration and Invasion Assay

Migration and invasion assays of the Control, CTS-treated (60 μM) and Stattic-treated (2 μM), and STAT3 knockdown cells (STAT3i-1415) were performed as described previously (22) and every group was repeated in triplicate. The detailed information was provided in the Supplementary Materials and Methods.

### Plate Clone Formation Assay

Cell proliferation was assessed by plate clone formation assay and the detailed information was provided in the Supplementary Materials and Methods.

### ELISA Measurement of Serum FGF3 Concentration in TA2 Mice With Different Number of Pregnancies

After standing for 30 min, whole blood samples from TA2 mice with different numbers of pregnancies were centrifuged for 15 min at 3,000 g to separate the serum. The serum levels of FGF3 were then determined using ELISA kits (Wuhan ColorfulGene Biological Technology, China).

### Reverse Transcription-Polymerase Chain Reaction (RT-PCR)

Total RNA from normal breast tissue in TA2 mice with different numbers of pregnancies and SBC was isolated using TRizol reagent (Invitrogen, USA) and reversely transcripted into cDNA according to the manufacturer's instructions (Novcare Biotech, China). The level of MMTV-LTR expression was normalized to that of glyceraldehyde 3-phosphate dehydrogenase (GAPDH). The MMTV-LTR primer sequences were 5′GACATGAAACAACAGGTACATGA3′ and 5′GGACTGTTGCAAGTTTACTC 3′ (full length 339 bp). The GAPDH primer sequences were 5′ACCACAGTCCATGCCATCAC3′ and 5′TCCACCACCCTGTTGCTGTA3′ (full length 452 bp).

### Western Blot Assay

Western blot analysis was performed as our previously described (23–25). Information about the primary antibodies and reagents is listed in the Supplementary Materials and Methods. Detailed information on the antibodies is listed in Supplementary Table 2. All these western blot assays were repeated three times.

### Immunocytochemical (ICC) Staining and Immunohistochemical (IHC) Staining

ICC and IHC staining were performed as previously described (7, 23). Detailed information, including the primary antibodies used, is provided in the Supplementary Materials and Methods and Supplementary Table 2.

### Animal Experiments

Twenty female TA2 mice (6 weeks of age, 20–25g) were randomly divided into the control (5 mice) vs. CTS (5 mice), and control (5 mice) vs. Stattic (5 mice) groups. The mice were injected subcutaneously on the right flank with MA-891 cells (1 × 10^6^ cells/mouse) suspended in 100 μL serum-free medium. The inhibitor groups were subcutaneously administered once every 2 days from the fifth day after inoculation. The concentrations of CTS and Stattic were 60 μM and 2 μM, respectively. Starting on the eighth day after injection, the tumors could be palpated and measured every 2 days. The tumor size was determined according to the formula: Tumor volume (mm^3^) = (length × width^2^)/2 and the tumor growth curve was plotted. On the 18th day after inoculation, all mice were sacrificed and the tumor tissues were removed and photographed. Fresh tumor tissues from each mouse were frozen for western blot analysis.

### Scoring of IHC Staining

For the scoring of immunostained tissue sections, both the intensity and percentage of positive cells were evaluated according to the methods described by Fei et al. (22, 24). The detailed information is shown in the Supplementary Materials and Methods.

### Statistical Analysis

SPSS Statistics for Windows, version 17.0 (IBM Corp., USA) was used to evaluate the data. One-way ANOVA was used to compare the differences in MMTV mRNA and protein expression. Kruskal–Wallis test was performed to compare the differences of the protein expression levels. Other comparisons were performed with two-tailed Student′s *t*-test and Pearson′ s chi-square (χ2) test. In this study, *P* < 0.05 was considered statistically significant.

## Results

### Morphological Features in Breast Tissue of TA2 With Different Number of Pregnancies and SBC

**Figure 1:**
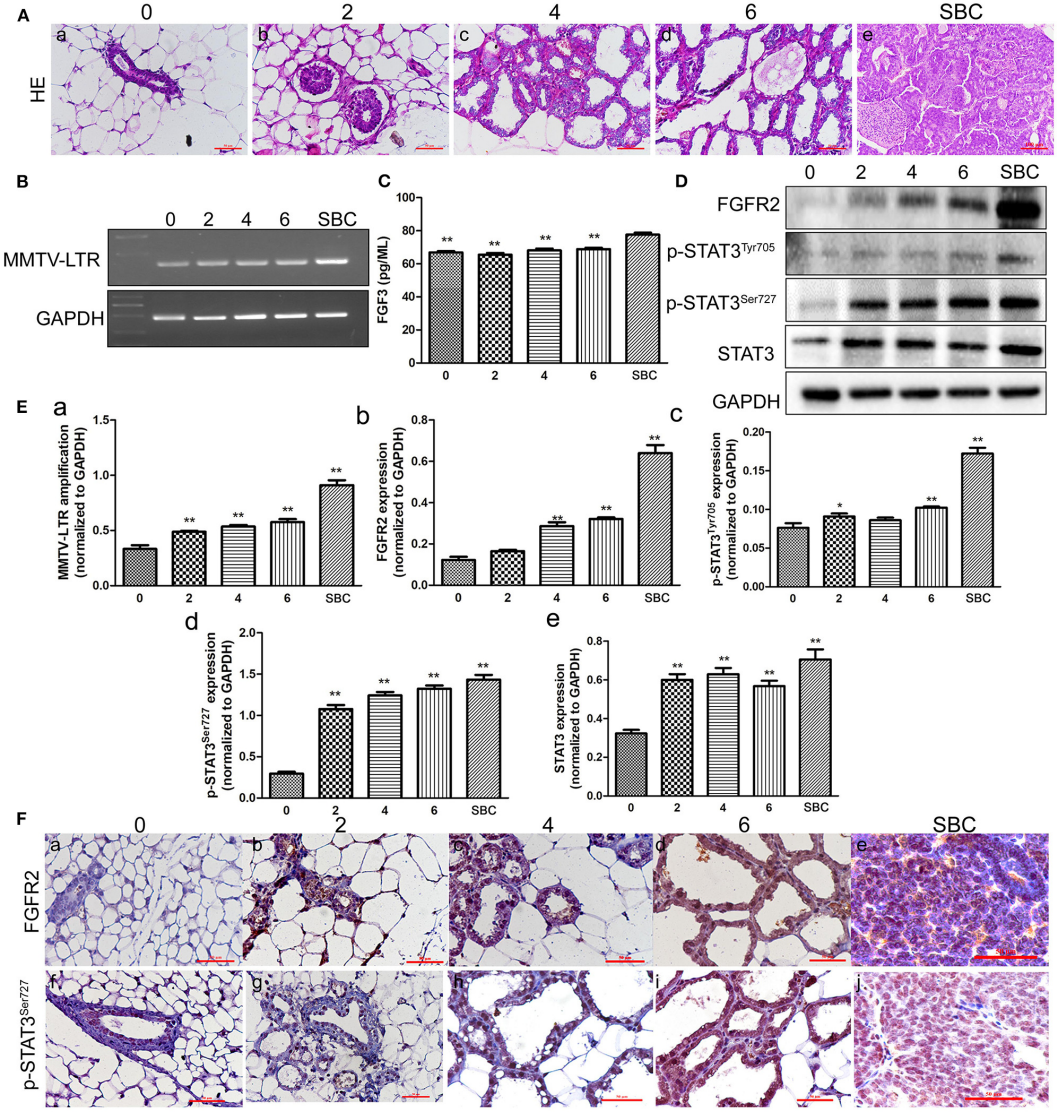
Expression of mouse mammary tumor virus-long terminal repeat (MMTV-LTR) and related proteins in the serum and breast tissue of Tientsin Albino 2 (TA2) mice with different numbers of pregnancies and spontaneous breast cancer (SBC). **(A) (**a–e) Morphological changes in the breast tissues in TA2 mice with zero, two, four, and six pregnancies and SBC, respectively (H&E,×40). **(B)** Results of reverse transcription-polymerase chain reaction (RT-PCR) analysis of MMTV-LTR from TA2 mice with no, two, four, and six pregnancies and SBC, respectively. **(C)** Serum FGF3 concentrations in TA2 mice with different numbers of pregnancies and SBC by enzyme-linked immunosorbent assay (ELISA). **(D)** Expression levels of fibroblast growth factor receptor 2 (FGFR2), phosphorylated signal transducer and activator of transcription 3 (p-STAT3)^Tyr705^, p-STAT3^Ser727^, and STAT3 by western blot analysis in TA2 mice with different numbers of pregnancies and SBC. **(E)** Histograms showing quantitative differences in MMTV-LTR and related protein expression in the serum and breast tissue of TA2 mice with different number of pregnancies and SBC. Each bar represents the mean ± standard deviation (SD) of three independent experiments. **(F)** Immunohistochemical staining of FGFR2 and p-STAT3^Ser727^ (IHC, ×40). (a–e). FGFR2 staining in breast tissues of TA2 mice with 0, 2, 4, and 6 pregnancies and with SBC, respectively. (f–j). p-STAT3^Ser727^ staining in breast tissues of TA2 mice with no, two, four, and six pregnancies and SBC, respectively. Statistically differences are indicated: **P < 0.001; *P < 0.05.

H&E staining of tissues including mammary glands of mice with different number of pregnancies and SBC showed gradually increased hyperplasia with increasing pregnancy. The structure of mammary duct and lobule is intact in the normal mammary glands of TA2 mice without a history of pregnancy (Figure 1Aa). Breast tissue from mice with two pregnancy times showed mild hyperplasia of the mammary gland epithelium, without obvious changes of the glandular tube (Figure 1Ab). Breast tissue of mice with four pregnancy times showed obvious hyperplasia of the mammary gland epithelium was obvious and slightly dilated glandular tube (Figure 1Ac). TA2 mice with six pregnancy times showed severe hyperplasia of the mammary gland epithelial cells and highly dilated duct. The shape of mammary glands was irregular and some were cystoid. The glands were also larger (Figure 1Ad). The tissue of SBC were composed of numerous solid tumor nests and glandular structures with few stroma (Figure 1Ae).

### Expression of FGFR2, STAT3, p-STAT3^Tyr705^, and p-STAT3^Ser727^ and MMTV-LTR Amplification in TA2 Mice With Different Number of Pregnancies and SBC

Our previous studies confirmed that the presence of MMTV was related to SBC initiation in TA2 mice (4, 5, 7). Moreover, there have been reports that MMTV indirectly promotes breast tumor formation by inserting into *FGF3* and *FGFR2* (14). To clarify the relationships among MMTV, FGFR2/STAT3, and SBC initiation, RT-PCR was used to detect MMTV-LTR expression in normal breast tissue from TA2 mice with different numbers of pregnancies and SBC. The results confirmed a significant increase in MMTV-LTR amplification in breast tissue with increasing number of pregnancies compared to the level in the breast tissue of TA2 mice without pregnancy; moreover, SBC had the highest expression of (Figures 1B,Ea). Furthermore, ELISA was used to detect the serum FGF3 level in the different groups and the results showed that the level of FGF3 in TA2 with SBC was the highest and the differences between TA2 with different number of pregnancies and SBC had significant significances (Figure 1C). No significant differences were observed in the serum levels of FGF3 among TA2 mice with and without pregnancy. Analysis of FGFR2, STAT3, p-STAT3^Tyr705^, and p-STAT3^Ser727^ expression by western blot of normal breast tissue with different number of pregnancies and SBC (Figure 1D) showed gradually increasing expression FGFR2, p-STAT3^Tyr705^, and p-STAT3^Ser727^ with increasing numbers of pregnancies. FGFR2, p-STAT3^Tyr705^ and p-STAT3^Ser727^ expression levels were highest in the SBC group. Compared to those in the breast tissue from TA2 mice without pregnancy, the expression of FGFR2 (Figure 1Eb), p-STAT3^Tyr705^ (Figure 1Ec), and p-STAT3^Ser727^ (Figure 1Ed) increased in TA2 mice with different numbers of pregnancies and SBC, with statistically significant differences (Figures 1Eb–d). Normal breast tissue and SBC had the lowest and highest STAT3 expression, respectively. There were no differences among breast tissue of TA2 mice with different numbers of pregnancies (Figure 1Ee). Furthermore, IHC showed that FGFR2 and p-STAT3^Ser727^ expression gradually increased in the breast tissue of TA2 with different number of pregnancies (Figures 1Fb–d,g–i) and SBC (Figures 1Fe,j) compared to that in normal breast tissue (Figures 1Fa,f), which was consistent with the western blot results.

### CTS and Stattic Inhibited STAT3, p-STAT3^Ser727^, p-STAT3^Tyr705^, Bcl2, Cyclin D1 and, c-myc Expression in MA-891 Cells

**Figure 2:**
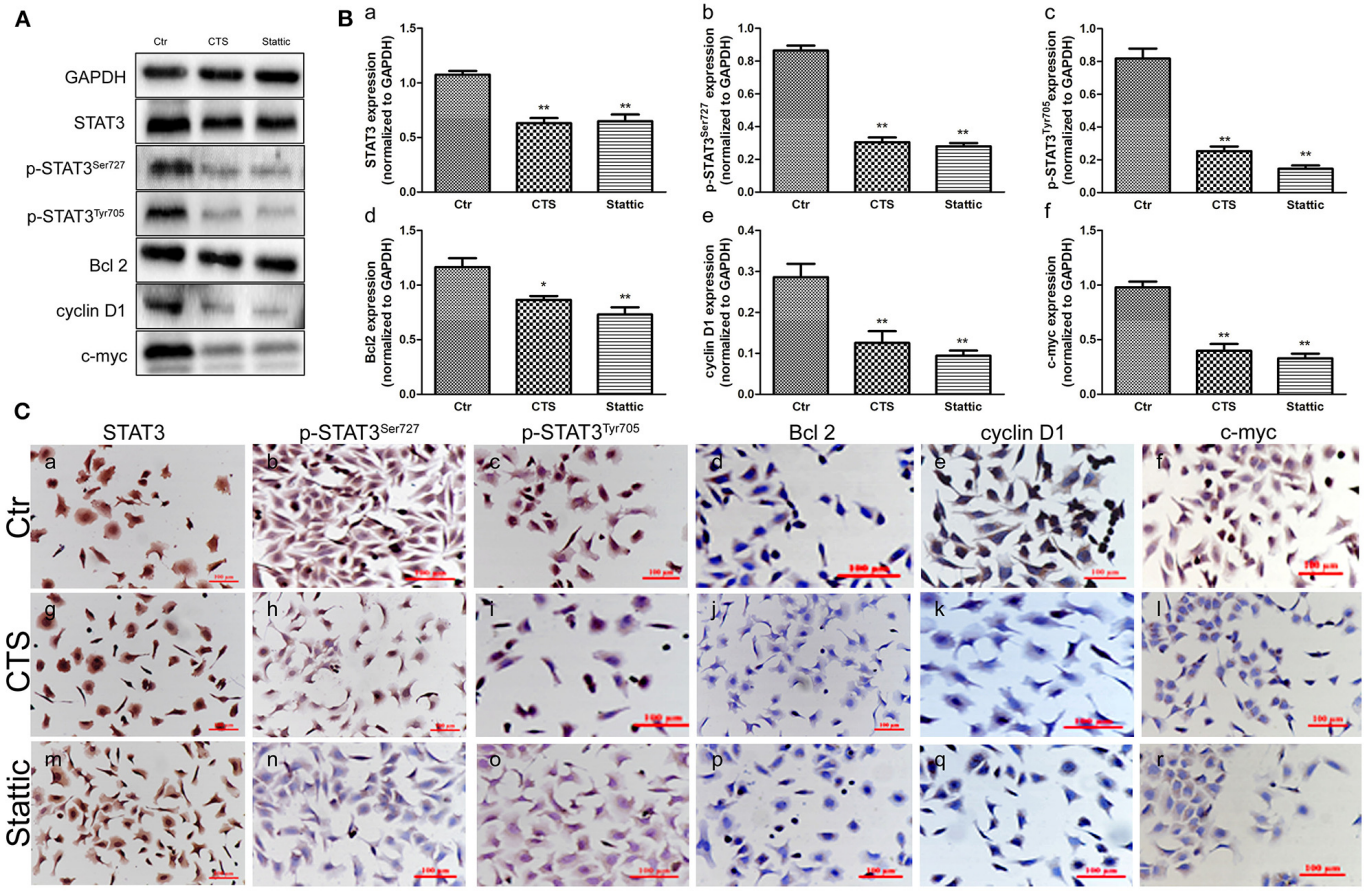
Protein expression in control and cryptotanshinone (CTS) and Stattic-treated MA-891 cells. **(A)** Western blot analysis showing STAT3, p-STAT3^Ser727^, p-STAT3^Tyr705^, Bcl2, cyclin D1, and c-myc expression in control, CTS-treated, and Stattic-treated cells. **(B)** Histograms of quantitative differences in protein expression among control, CTS and Stattic-treated MA-891 cells. Each bar represents the mean ± standard deviation (SD) of three independent experiments. **(C)** Immunocytochemical (ICC) staining of STAT3, p-STAT3^Ser727^, p-STAT3^Tyr705^, Bcl2, cyclin D1, and c-myc. (a–f). STAT3, p-STAT3^Ser727^, p-STAT3^Tyr705^, Bcl2, cyclin D1 and c-myc staining in control MA-891 cells, respectively. (g–l). STAT3, p-STAT3^Ser727^, p-STAT3^Tyr705^, Bcl2, cyclin D1, and c-myc staining in the CTS-treated group, respectively. (m–r). STAT3, p-STAT3^Ser727^, p-STAT3^Tyr705^, Bcl2, cyclin D1 and c-myc in staining in the Stattic-treated group, respectively. Statistically differences are indicated: **P < 0.001; *P < 0.05.

To study the mechanisms of STAT3 in the initiation of TA2 SBC, MA-891 cells derived from SBC of TA2 mice were treated with two kinds of STAT3 small molecule inhibitors (CTS and Stattic). Western blot showed that CTS and Stattic inhibited STAT3, p-STAT3^Ser727^, p-STAT3^Tyr705^, Bcl2, cyclin D1, and c-myc expression (Figure 2A), with significant differences in expression between the treatment and control groups for STAT3 (Figure 2Ba), p-STAT3^Ser727^ (Figure 2Bb), p-STAT3^Tyr705^ (Figure 2Bc), Bcl2 (Figure 2Bd), cyclin D1 (Figure 2Be), and c-myc (Figure 2Bf). In addition, the expression and subcellular localization of these proteins was also observed in the MA-891 cells before and after CTS and Stattic treatments by ICC (Figure 2C). In control MA-891 cells, STAT3, p-STAT3^Ser727^, and p-STAT3^Tyr705^ were mainly located in the nucleus. Cyclin D1 was located in the nucleus to play a role in cell proliferation. Bcl-2, which can inhibit apoptosis, was mainly located in the cytoplasm while c-myc was mainly located in the nucleus. After treatment with CTS and Stattic, no obvious changes in the localization and expression of total STAT3 were observed (Figures 2Ca,g,m). However, the expression of p-STAT3^Ser727^ (Figures 2Cb,h,n) and p-STAT3^Tyr705^ (Figures 2Cc,i,o) decreased compared to those the control cells. Additionally, compared to the control cells, the expression of Bcl2 (Figures 2Cd,j,p), cyclin D1 (Figures 2Ce,k,q), and c-myc (Figures 2Cf,l,r) also decreased in MA-891 cells after treatment.

### Down-Regulation of STAT3 Expression Inhibited MA-891 Cell Proliferation

**Figure 3:**
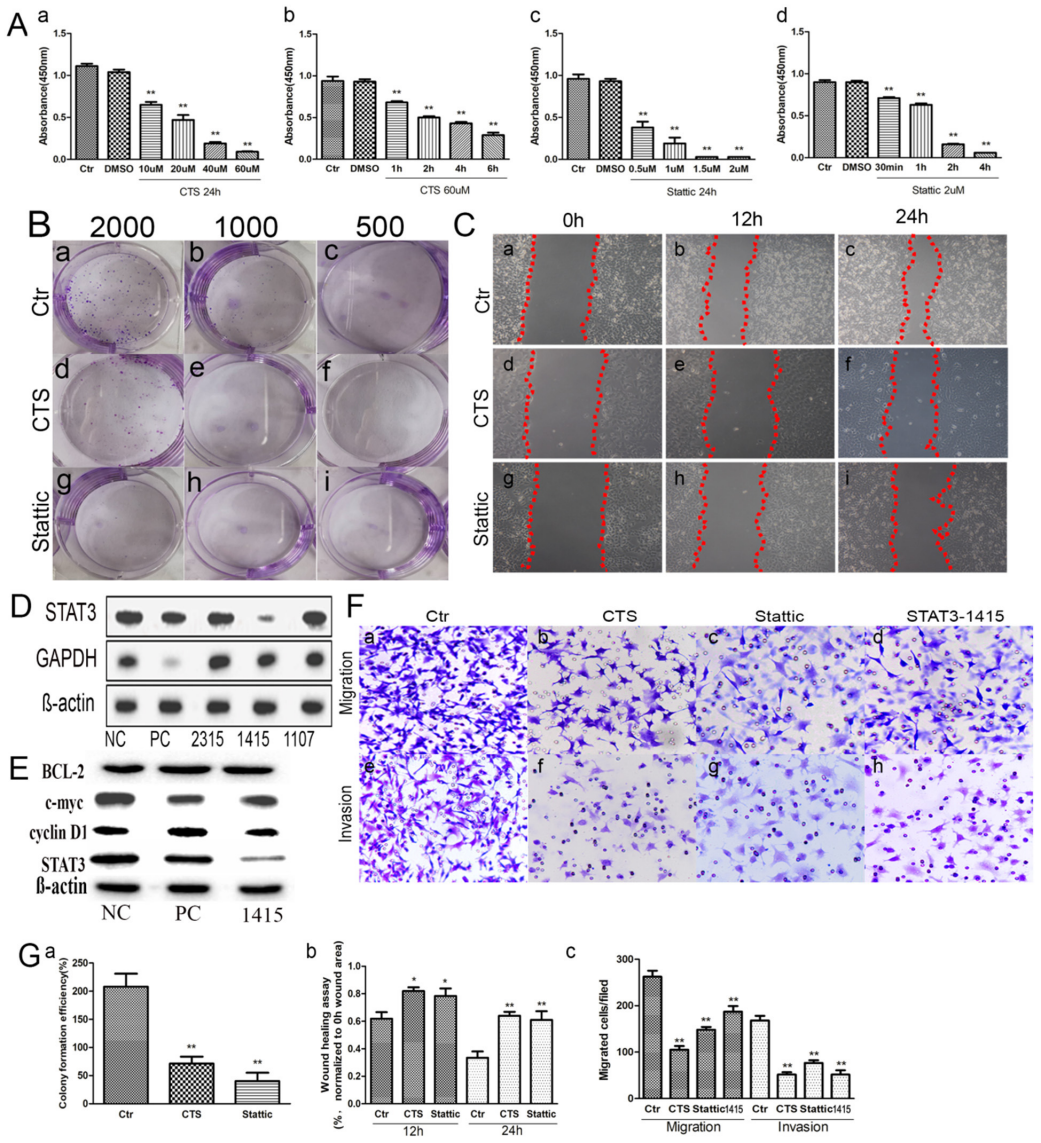
Comparisons of proliferation, migration, and invasive ability in control, cryptotanshinone (CTS)-treated, and Stattic-treated MA-891 cells. **(A)** Cell counting kit-8 (CCK8) shows that CTS and Stattic inhibit viability in a time- and dose-dependent manner. (a) Comparisons of viability in control cells and cells treated with different concentrations of CTS for 24 h. (b) Comparisons of viability in control cells and cells treated with 60 μM of CTS for different times. (c) Comparisons of viability in control cells and cells treated with different Stattic concentrations for 24 h. (d) Comparisons of viability in control cells and cells treated with 2 μM Stattic for different time. **(B)** Cell proliferation ability based on clone formation. (a–c) Proliferation ability of 2,000, 1,000, and 500 control cells, respectively. (d–f) Proliferation ability of 2,000, 1,000, and 500 cells after CTS treatment, respectively. (g–i) Proliferation ability of 2,000, 1,000, and 500 cells after Stattic treatment, respectively. **(C)** Wound-healing assay in MA-891 cells at 0, 12, and 24 h, respectively, after different treatments. (a–c) Representative images in control cells. (d–f) Representative images after CTS treatment. (g–i) Representative images after Stattic treatment. **(D)** Western blot showed STAT3 and GAPDH expression in MA-891 cells with siRNA STAT3-2315, 1415, 1107, positive control, and negative control. **(E)** Western blot showed STAT3, cyclin D1, c-myc and Bcl2 expression in MA-891 cells with siRNA STAT3-1415, positive control, and negative control. **(F)** Transwell migration and invasion assay in MA-891 cells before and after treatment. (a–d) Migration ability in control, CTS-treated, Stattic-treated cells, and cells after STAT knockdown. (e–h) Invasion assay of control, CTS-treated, Stattic-treated cells and cells after STAT knockdown. **(G)** Histograms showing the quantitative results of the proliferation, migration, and invasive ability of MA-891 cells before and after treatment. Each bar represents the mean ± standard deviation (SD) of three independent experiments. Statistically differences are indicated: **P < 0.001; *P < 0.05. 1415: siRNA STAT3-1415. PC, positive control; NC, negative control.

To determine the effect of small molecular inhibitors on MA-891 cell viability, we carried out CCK8 detection after CTS and Stattic treatment. After 24 h incubation, the absorbance of the control and treated cells was measured (Figure 3A), showing dose-dependent effects of the two inhibitors on cell viability. The inhibitory effect on the viability of MA-891 cells increased with the concentration of CTS and Stattic. Compared to the control cells, the differences for DMSO, 10, 20, 40, and 60 μM CTS and for DMSO, 0.5, 1, 1.5, and 2 μM Stattic are shown in Figures 3Aa,c, respectively. The inhibitory effect on MA-891 cells with 60 μM CTS and 2 μM Stattic increased with the treatment time. Compared to the control cells, the differences for DMSO, 1, 2, 4, and 6 h of CTS and for DMSO, 30 min, 1, 2, and 4 h Stattic are shown in Figures 3Ab,d. Moreover, plate cloning assay was used to assess cell proliferation ability, with 2,000 (Figures 3Ba,d,g), 1,000 (Figures 3Bb,e,h), and 500 (Figures 3Bc,f,i) control, CTS-treated, and Stattic-treated MA-891 cells seeded in six-well plates, respectively. The results showed that CTS and Stattic inhibited clone formation. The proliferation ability in the control group was significantly higher than those in the CTS and Stattic treatment groups (Figure 3Ga).

### Down-Regulation of STAT3 Expression Inhibited MA-891 Cell Migration and Invasion

To determine whether CTS and Stattic treatment affected cell migration in MA-891 cells, wound healing and transwell migration assays were performed using control, CTS-treated, and Stattic-treated cells. Figure 3C shows the results of the wound-healing assays at 0, 12, and 24 h. The areas covering the scratched surface gradually decreased with the extension of incubation periods (Figures 3Ca–i). Cell migration in the CTS-treated and Stattic-treated cells were decreased at 12 and 24 h compared to that at 0 h (Figure 3Gb). Moreover, results of the transwell migration assay confirmed a higher number of migrating cells for control MA-891 cells than for CTS-treated and Stattic-treated cells. To examine whether CTS and Stattic treatment affected cell invasion in MA-891 cells, we performed cell invasion assays using transwell assay with matrigel-coated inserts. The numbers of invading cells were markedly lower in the CTS and Stattic treatment groups compared to those in the control cells (Figures 3Fa–c,e–g). In order to avoid the off-targets effects, MA-891 cells after STAT3 knockdown were used to measure the migration and invasion ability of cells. Results of western blot showed that siRNA STAT3-1415 had the strongest inhibitory efficiency and was used in this study (Figure 3D). In addition, the expression of cyclin D1 and c-myc decreased in MA-891 cells after siRNA STAT3-1415 treatment compared to the negative control and positive control. However, there were not obvious differences of Bcl2 expression among siRNA STAT3-1415, negative control and positive control MA-891 cells (Figure 3E). After STAT3 was inhibited, the numbers of migrating and invasive cells also decreased compared to those in the control cells (Figures 3Fd,h). Quantitative assessment of the transwell invasion assays showed significant differences of the invasive cell numbers for CTS, Stattic treatment and STAT3 knockdown (Figure 3Gc).

### STAT3 Expression in TA2 SBC Tissues Promoted Tumor Growth

**Figure 4:**
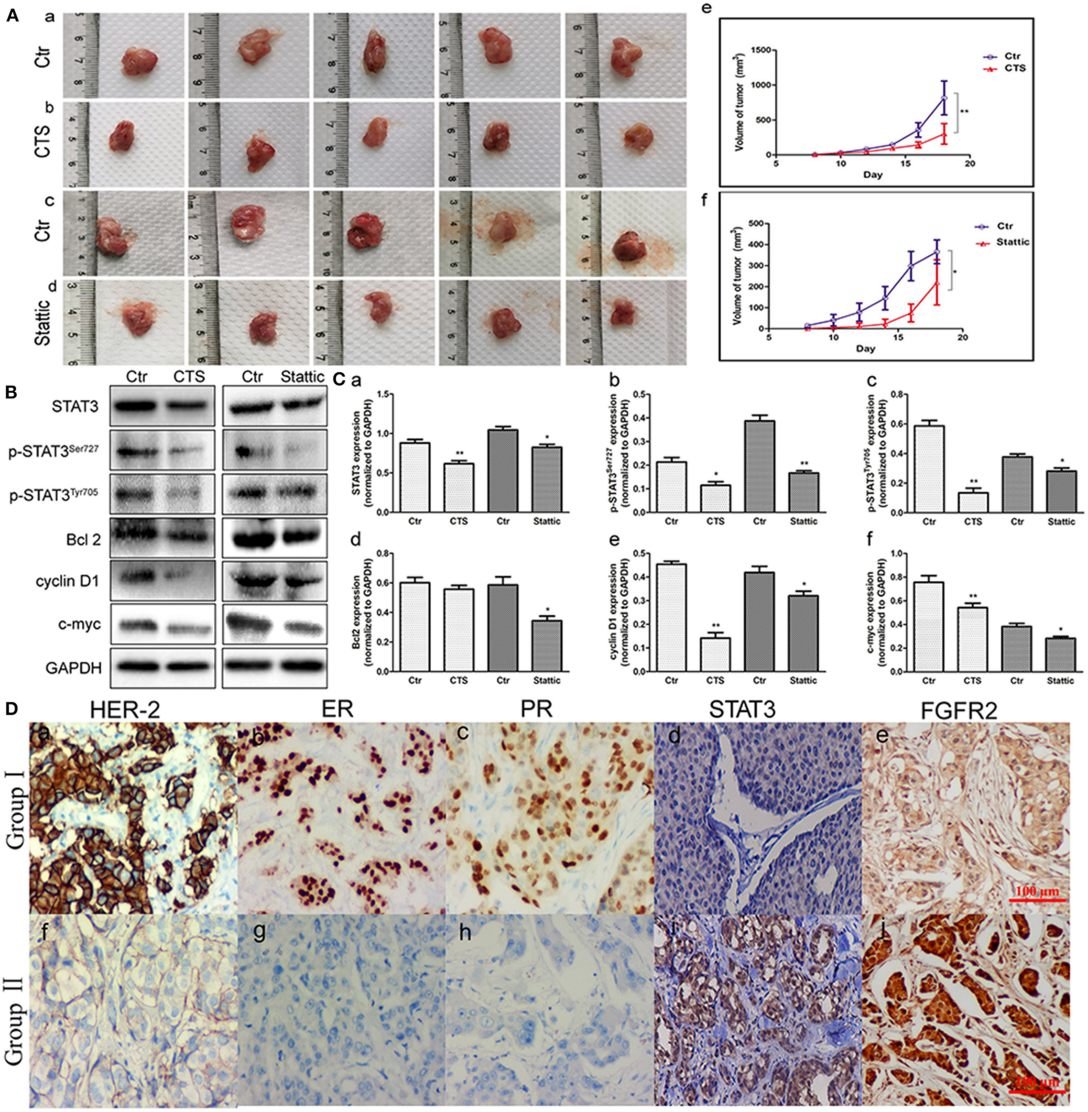
Fibroblast growth factor receptor 2/signal transducer and activator of transcription 3 (FGFR2/STAT3) signaling pathway-related proteins associated with the development of animal xenografts from MA-891 and human breast cancer. **(A)** Gross pictures of tumor masses (each tumor was from one mouse) after the 18th day. (a–d) Tumor tissue of TA2 xenografts from control MA-891 cell injection (a), control MA-891 cell injection and cryptotanshinone (CTS) administration (b), control MA-891 cell injection (c), and control MA-891 cell injection and Stattic administration, respectively (d). (e–f) Growth curves of animal xenografts from control and CTS administration (e), control and Stattic administration (f), respectively. **(B)** Western blot analysis of the expression of proteins including STAT3, p-STAT3^Ser727^, p-STAT3^Tyr705^, Bcl2, cyclin D1, and c-myc in xenografts with control and CTS and Stattic treatments. **(C)** Histograms of quantitative differences in protein expression. Each bar represents the mean ± standard deviation (SD) of three independent experiments. **(D)** Immunohistochemical (IHC) staining of human epidermal growth factor receptor 2 (HER-2), estrogen receptor (ER), progesterone receptor (PR), and STAT3 in human breast cancer tissues (IHC × 100). (a–c) HER-2-, ER-, and PR-positive breast cancer. (d) STAT3 expression in non-TNBC. (e) FGFR2 expression in non-TNBC. (f) HER-2-negative breast cancer. (g) ER-negative breast cancer. (h) PR-negative breast cancer. (i) STAT3 expression in TNBC. (j) FGFR2 expression in TNBC. Statistically differences are indicated: **P < 0.001; *P < 0.05.

MA-891 cells derived from TA2 SBC were subcutaneously injected into the right flank of TA2 mice to investigate the effect of STAT3 on xenograft growth and proliferation. From the fifth day after injection, CTS and Stattic were administered to mice with xenografts once every 2 days (four times in total). Eight days after injection, xenografted tumors could be palpated on the flanks of the mice. All mice were sacrificed on the 18th day after injection and the tumor tissues were removed (Figures 4Aa–d). The tumor lengths and widths were measured and the volumes were determined. As shown in Figure 4A, the average xenograft volume in the CTS and Stattic treatment groups was significantly lower than it in the control group, with statistically significant differences between the two groups (Figures 4Ae,f).

### Expression of FGFR2/STAT3 Signaling Pathway-Related Proteins in TA2 Xenografts Before and After CTS and Stattic Treatment

Proteins were extracted from tumor tissues in the four groups of animal xenografts for western blot analysis. The results showed that CTS and Stattic inhibited the expression of STAT3, p-STAT3^Ser727^, p-STAT3^Tyr705^, Bcl2, cyclin D1, and c-myc (Figure 4B). Compared to the control, the protein expression levels of STAT3, p-STAT3^Ser727^, p-STAT3^Tyr705^, Bcl2, cyclin D1, and c-myc were lower in the Stattic and CTS-treated groups (Figures 4Ca–f), indicating that STAT3 might be important for tumor development and progression in TA2 SBC.

### STAT3 and FGFR2 Expression in Human Breast Cancer Tissues

To measure the STAT3 and FGFR2 expression levels and their clinicopathological significance, 139 cases of human breast cancer tissue including 117 non-TNBC and 22 TNBC were subjected to IHC staining for STAT3. The average STAT3 and FGFR2 staining index in the TNBC group (group II) were higher than them in the non-TNBC group (group I) and the difference had statistical significance (Figure 4D) (Table 1).

**Table 1:** Differences of the average STAT3 and FGFR2 staining index between non-TNBC and TNBC.

		**Group**	**Number**	**Average staining index**	**Z**	***P***
STAT3	Non-TNBC	I	117	3.299 ± 2.00	−4.265	0.001
	TNBC	II	22	5.50 ± 2.22		
FGFR2	Non-TNBC	I	117	3.94 ± 1.23	−4.230	0.001
	TNBC	II	22	5.23 ± 1.07		

## Discussion

The TA2 mouse model, an ideal animal model for breast cancer research, has a high incidence of SBC. Our previous studies showed the tumorigenesis of TA2 SBC was estradiol and progesterone-dependent. High levels of estradiol and progesterone during pregnancy bind to the HRE of MMTV-LTR to promote the amplification of MMTV and induce the initiation of SBC in TA2 mice (7). MMTV usually integrates into *Wnt, fgf, fgfr, Rspo*, and *Pdgfr*-related locus sites, which contribute to carcinogenic protein amplification. Previous studies have shown that *Wnt* and *fgf3* insertion sites play important roles in TA2 SBC, consistent with our previous findings that Wnt/β-catenin is involved in SBC development and progression in TA2 mice (5).

FGFs and FGFRs are involved in different physiological processes and tumor development associated with proliferation, survival, differentiation, migration, and apoptosis by activating the STAT3, MAPK, and other pathways (26, 27). FGF3, a member of the FGF family, regulates several processes including brain developmental pattern, and limb development by binding, and activating FGFRs in cell surface (28). FGF3/FGFRs are also associated with cellular proliferation, infiltration, and invasiveness during the initiation and development of cancer (29, 30). FGFR1, FGFR2, FGFR3, and FGFR4 are the members of receptor tyrosine kinase subfamily (31). FGF/FGFR2 signaling axis plays an important role in the development of breast cancer. Single nucleotide polymorphisms in intron 2 of the FGFR2 gene are associated with the incidence of breast cancer (32–34).

TNBC is a subtype of invasive breast cancer with ER, PR, and HER-2 negativity that accounts for approximately 15–20% of all breast carcinomas (35, 36). TNBC is also more likely to occur in childbearing women and the prognosis of patients was poor (37). Recent studies have shown that abnormal STAT3 expression plays a vital role in TNBC (12). Similar to FGFR2, STAT3 can also promote tumor growth. In static-phase cells, STAT3 remains in the cytoplasm at its inactive form. Once phosphorylated and activated, STAT3 can translocate to the nucleus to provide transcriptional activity for specific target genes including Bcl2, cyclin D1, c-myc, etc. Upon its activation, this transcription factor regulates malignant tumor proliferation, survival, and metastasis (11, 38). The activity of STAT3 increases in TNBC and regulates proliferation, metastasis, and radiochemotherapy resistance, suggesting that targeting of STAT3 signaling might be an effective therapy in TNBC (38, 39).

This study measured the expression of FGFR2/STAT3 signaling pathway-related proteins and the amplification of MMTV-LTR in TA2 mice with different number of pregnancies and SBC. The results showed gradually increasing breast tissue expression of FGFR2, p-STAT3^Ser727^, and p-STAT3^Tyr705^ with increasing number of pregnancies and highest expression in SBC, a finding consistent with the amplification of MMTV-LTR. The expression of total STAT3 in mice with a history of pregnancy and SBC was higher than that in mice without a history of pregnancy. Furthermore, the serum FGF3 expression in SBC was higher than those in TA2 mice with different numbers of pregnancies. Third, to determine the role of the FGFR2/STAT3 signaling pathway in the development of SBC in TA2 mice, two kinds of STAT3 small molecule inhibitors were used to inhibit STAT3 expression and phosphorylation in MA-891 cells and the mouse xenograft model. The results showed decreased proliferation, migration, and invasion in MA-891 cells after treatment. Meanwhile, the average volumes of xenografts in the CTS and Stattic treatment groups were significantly decreased compared to that in the control group. The expression of important downstream proteins of the FGFR2/STAT3 signaling pathway including Bcl2, cyclin D1, and c-myc decreased in MA-891 cells and animal xenografts after CTS and Stattic treatment. CST, a natural product isolated from Salvia miltiorrhiza Bunge, can significantly inhibit the STAT3^Tyr705^ phosphorylation and the dimerization of STAT3 (40). Stattic can inhibit STAT3^Tyr705^ and STAT3^Ser727^ phosphorylation (41). When STAT is phosphorylated and forms dimerization, it can translocate into the nucleus and become a transcription factor. However, in the resting cells, STAT3 retains in the cytoplasm. In this study, results of western blot showed that CTS and Stattic inhibited STAT3, p-STAT3^Ser727^, p-STAT3^Tyr705^ expression. In addition, CTS and Stattic could also inhibit the expression of basal STAT3. We speculate that basal STAT3 accumulated into the cytoplasm after the phosphorylation inhibited by CTS and Stattic. The cytoplasmic STAT3 expression might be regulated by the upstream proteins and decreased via negative feedback. In order to avoid the off-targets effects, siRNA was used to inhibit the expression of STAT3 and the ability of migration and invasion of MA-891 decreased after STAT3 knockdown. Fourth, we also measured STAT3 and FGFR2 expression in human breast tissue, observing significantly higher expression in TNBC than them in non-TNBC. These results indicate that the FGFR2/STAT3 signaling pathway may promote SBC initiation in TA2 mice.

In summary, the results of the present study provide evidence that FGF3 and FGFR2 expression and STAT3 phosphorylation are associated with the number of pregnancies, which increased the occurrence of SBC in TA2 mice. Inhibition of STAT3 can decrease proliferation, invasiveness, and migration in MA-891 cells and the growth of TA2 xenografts. As a key transcription factor, STAT3 may be a potential therapeutic target for patients with TNBC. Based on our previous studies, the high incidence of TA2 SBC associated with the gravidity, the frequency of pregnancy, and presence of MMTV. The detail molecular mechanism of TA2 spontaneous breast cancer is very complex and more experiments are needed to confirm the relationship between FGFR2/STAT3 signaling pathway and the tumorigenesis in TA2 mice in the future.

## Data Availability Statement

The raw data supporting the conclusions of this article will be made available by the authors, without undue reservation, to any qualified researcher.

## Ethics Statement

The studies involving human participants were reviewed and approved by Hospital Review Board of Tianjin Union Medicine Center. Written informed consent for participation was not required for this study in accordance with the national legislation and the institutional requirements. The animal study was reviewed and approved by Institutional Animal Care and Use Committee of Tianjin Union Medicine Center.

## Author Contributions

SZ designed the study and interpreted data contributed to manuscript writing and approved the manuscript before submission. JD, KL, and QZ collected and analyzed data and approved the manuscript before submission. ZL, FF and KZ collected, analyzed, and interpreted the data, and approved the manuscript before submission. HZ, MZ, and YZ collected data, gave constructive comments on the manuscript, and approved the manuscript before submission.

## Conflict of Interest

The authors declare that the research was conducted in the absence of any commercial or financial relationships that could be construed as a potential conflict of interest.

## Abbreviations

TA2: Tientsin Albino 2
STAT3: signal transducer and activator of transcription 3
MMTV: mouse mammary tumor virus
SBC: spontaneous breast cancer
FGF: Fibroblast growth factor
FGFR: fibroblast growth factor receptors
LTR: longer terminal repeats
ER: estrogen receptor
PR: progesterone receptor
TNBC: triple-negative breast cancer
CTS: cryptotanshinone
PBS: phosphate-buffered solution.
