# Correction: FGFR2/STAT3 signaling pathway involves in the development of MMTV-related spontaneous breast cancer in TA2 mice

**DOI:** 10.3389/fonc.2026.1837337

**Published:** 2026-05-13

**Authors:** Jiaxing Du, Qi Zhao, Kai Liu, Zugui Li, Fangmei Fu, Kexin Zhang, Hao Zhang, Minying Zheng, Yongjie Zhao, Shiwu Zhang

**Affiliations:** 1 Graduate School, Tianjin University of Traditional Chinese Medicine, Tianjin, China; 2 Department of Pathology, Tianjin Union Medical Center, Tianjin, China; 3 Graduate School, Tianjin Medical University, Tianjin, China; 4 Nankai University School of Medicine, Nankai University, Tianjin, China; 5 Departments of General Surgery, Tianjin Union Medical Center, Tianjin, China

**Keywords:** tientsin albino 2, FGFR2/STAT3, triple-negative breast cancer, spontaneous breast cancer, MMTV

There was a mistake in **Figure 3** as published. **Figure 3B** (c) and **Figure 3B** (g) are duplicates. **Figure 3B** (e) and **Figure 3B** (f) are also duplicates. The corrected **Figure 3** appears below.

**Figure 3:**
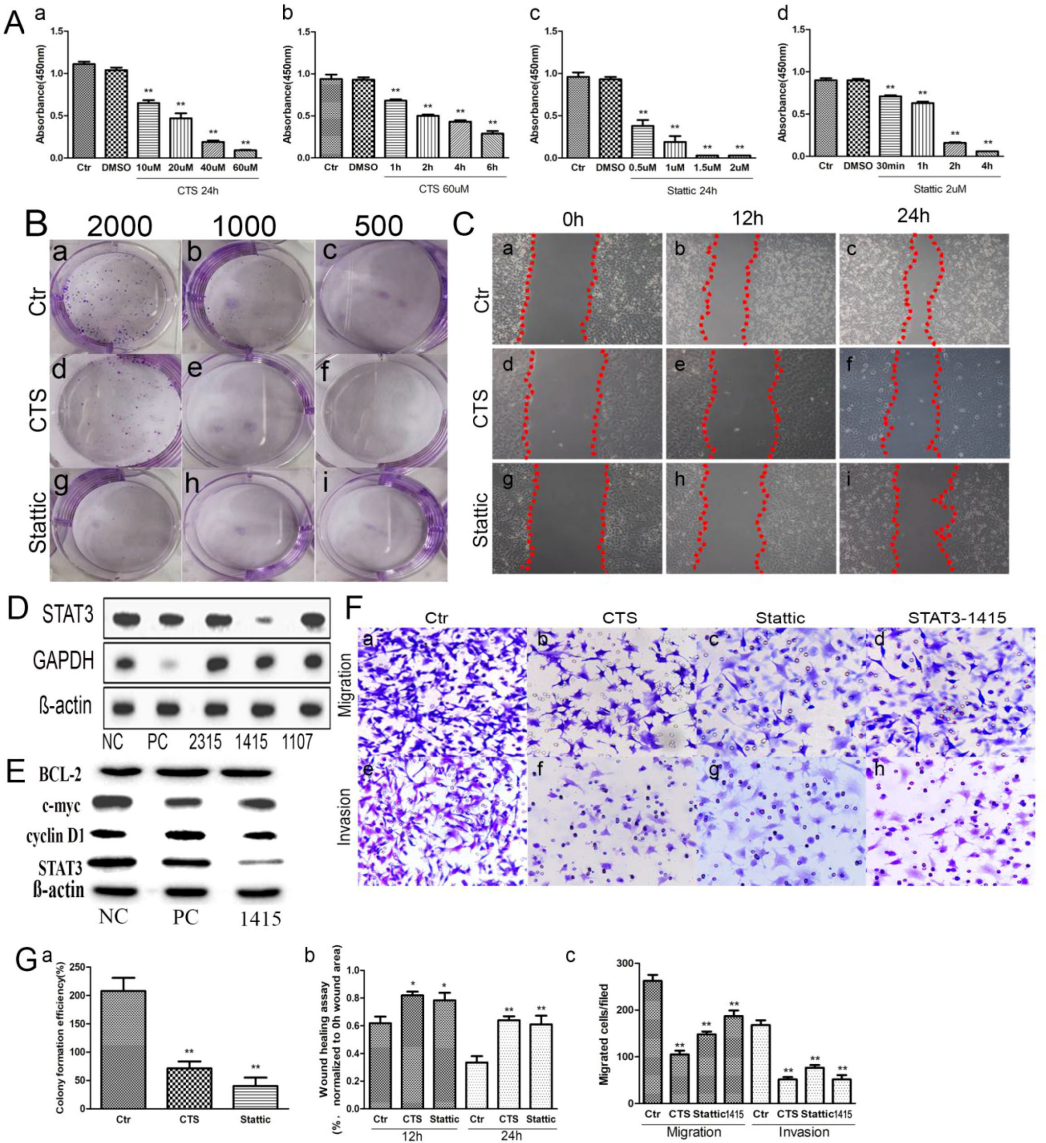
Comparisons of proliferation, migration, and invasive ability in control, cryptotanshinone (CTS)-treated, and Stattic-treated MA-891 cells. **(A)** Cell counting kit-8 (CCK8) shows that CTS and Stattic inhibit viability in a time- and dose-dependent manner. (a) Comparisons of viability in control cells and cells treated with different concentrations of CTS for 24 h. (b) Comparisons of viability in control cells and cells treated with 60 μM of CTS for different times. (c) Comparisons of viability in control cells and cells treated with different Stattic concentrations for 24 h. (d) Comparisons of viability in control cells and cells treated with 2 μM Stattic for different time. **(B)** Cell proliferation ability based on clone formation. (a–c) Proliferation ability of 2,000, 1,000, and 500 control cells, respectively. (d–f) Proliferation ability of 2,000, 1,000, and 500 cells after CTS treatment, respectively. (g–i) Proliferation ability of 2,000, 1,000, and 500 cells after Stattic treatment, respectively. **(C)** Wound-healing assay in MA-891 cells at 0, 12, and 24 h, respectively, after different treatments. (a–c) Representative images in control cells. (d–f) Representative images after CTS treatment. (g–i) Representative images after Stattic treatment. **(D)** Western blot showed STAT3 and GAPDH expression in MA-891 cells with siRNA STAT3-2315, 1415, 1107, positive control, and negative control. **(E)** Western blot showed STAT3, cyclin D1, c-myc and Bcl2 expression in MA-891 cells with siRNA STAT3-1415, positive control, and negative control. **(F)** Transwell migration and invasion assay in MA-891 cells before and after treatment. (a–d) Migration ability in control, CTS-treated, Stattic-treated cells, and cells after STAT knockdown. (e–h) Invasion assay of control, CTS-treated, Stattic-treated cells and cells after STAT knockdown. **(G)** Histograms showing the quantitative results of the proliferation, migration, and invasive ability of MA-891 cells before and after treatment. Each bar represents the mean ± standard deviation (SD) of three independent experiments. Statistically differences are indicated: **P < 0.001; *P < 0.05. 1415: siRNA STAT3-1415. PC, positive control; NC, negative control.

The original version of this article has been updated.

