# Supplementary material for: FGFR2/STAT3 Signaling Pathway Involves in the Development of MMTV-Related Spontaneous Breast Cancer in TA2 Mice
Source: Front Oncol. 2020 May 5;10:652. doi: 10.3389/fonc.2020.00652 (PMC7214838; doi:10.3389/fonc.2020.00652)
Supplement: Supplementary file 1 [file Data_Sheet_1.doc]

**Supplementary materials and methods**

**Wound‑healing assay**

MA-891 cells (5×10^4^ per well) were seeded onto six-well plates and cultured for 36–48 h until 95% confluency. After that, these cells were treated with CTS (60 μM) and Stattic (2 μM). After treatment, the control and treated cells were uniformly scratched using sterile pipette tips. The detached cells were removed by washing with phosphate-buffered solution (PBS), followed by incubation in serum-free medium. The scratched area between the red-dashed regions was measured using Image-J software. The cells were photographed in marked areas at 0, 12, and 24 h using a microscope. Every group was repeated in triplicate.

**Cell viability cell counting kit-8 (CCK8) assay**

MA-891 cells were seeded at 2,000 cells per well in 96-well plates and incubated for 12 h. These cells were divided into groups and every group was repeated in triplicate. The cells were treated with CTS and Stattic at different concentrations and times. Twenty-four hours later, 10 µL of CCK8 (Dojndo, Japan) was added to each well and incubated for 2 h, including three control wells containing medium alone which were used as blanks for absorbance readings. These wells were detected using a Bio-Rad microplate reader at a wavelength of 450nm. The optical density (OD) was calculated as the average value of readings after subtracting the average value of the blank.

**Tumor cell migration and invasion assay**

Control, CTS-treated (60 μM) and Stattic-treated (2 μM), and STAT3 knockdown cells (STAT3i) were washed three times with FBS-free medium and counted using an automated cell counter (Invitrogen, CA, USA). Cell migration and invasive ability was performed by the transwell migration and invasion assay (8 μm; Corning Inc.) , respectively. For migration assay, Cells (5×10^4^ cells per insert) in 200 μL medium without FBS were added to the upper chamber. For invasion assay, Cells (2×10^5^ cells per insert) in 200 μL medium without FBS were added to the upper chamber. At the same time, culture medium containing 20% FBS was added to the lower chamber and the 24-well plates were incubated for 14 h at 37°C. Then the cells were fixed in methanol for 30 min and stained with 0.1% crystal violet for 30 min. The migration and invasion ability was assessed by counting the number of cells per field. Photos were taken at 100×magnification, and cells were counted from at least 5 different fields. Three independent experiments were performed.

**Plate clone formation** **assay**

Control cells and cells treated with CTS (60 μM) and Stattic (2 μM) were counted with a cell counter. Afterward, cell suspensions with 2,000, 1,000, and 500 cells per well were seeded into six-well plates and cultured for up to 14 days until colonies were clearly visible. The colonies were fixed with 4% paraformaldehyde, stained with 0.5% crystal violet, and counted. Colonies with more than 50 cells were counted.

**Western blot assay**

MA-891 cells and tissue samples including those from the mammary glands mice with different numbers of pregnancies, SBC, and tumors derived from MA-891 xenografts were harvested and lysed with a radioimmunoprecipitation assay (RIPA) buffer supplemented with a proteinase inhibitor cocktail. The protein concentrations were determined and the proteins were separated on 8% or 10% sodium dodecyl sulfate (SDS) polyacrylamide gels and transferred to polyvinylidene difluoride (PVDF) membrane (GE, USA). The membrane was blocked with 5% non-fat dry milk in tris-buffered saline with 0.1% Tween 20 (TBST) buffer for 2 h at room temperature and incubated with the corresponding primary antibodies for 14–16 h. The membranes were then incubated with secondary antibodies at room temperature for 2 h. Protein expression was finally detected using a ChemiDoc imaging system (BioRad, USA). The densitometric analyses of protein bands were performed using ImageJ software. Detailed information on the antibodies is listed in Supplementary Table-1.

**Immunocytochemical (ICC) staining, Immunohistochemical (IHC) staining and H&E staining**

For ICC staining, MA-891 cell suspensions were cultured on sterile coverslips in six-well plates. 1.5 ml medium was added into the well and cultured for 24 h in a CO_2_ incubator. The cells were treated with CTS (60 μM) for 2 h or Stattic (2 μM) for 4 h. After treatment, the cells were fixed with ice-cold methyl alcohol for 30 minutes and then rinsed with PBS. The cells were then blocked with endogenous peroxidase inhibitor (Zhongshan, Inc., China) and subsequently with goat serum (Zhongshan, Inc., China) for 15 and 20 min at room temperature. These cells were incubated with the primary antibodies at 4°C for 16 h, the secondary antibodies for 20 minutes, and horseradish peroxidase-labeled streptomycin (Zhongshan Inc, China) for 15 minutes. Finally, the slides were stained with 3, 3'-diaminobenzidine (DAB, Zhongshan Inc., Beijing, China) for 1–5 min, and counterstained with hematoxylin. For IHC staining, four-micrometer sections of formalin-fixed, paraffin-embedded engrafted animal tissues and human breast tissue were deparaffinized in xylene, dehydrated in a series of graded ethanol solutions, and heated for 2 minutes in a citrate buffer solution using an autoclave to block endogenous peroxidase activity. The tissue sections were incubated overnight at 4°C with the primary antibodies. Finally, the samples were stained with 3,3'-diaminobenzidine (DAB; Zhongshan, Inc., China) for 1–5 minutes and counterstained with hematoxylin. For H&E staining, after dehydration, the slides were first dyed with hematoxylin (Baso, China) for approximately 20 seconds and eosin for 20-30 seconds. Finally, the cells were dehydrated with absolute ethyl alcohol.

**Scoring of immunostained tissue sections**

The expression of STAT3 and FGFR2 in the tissue samples was evaluated and quantified based on the percentage of positive cells and staining intensity. Staining in the cytoplasm and nucleus was considered positive expression and PBS was used to replace the primary antibody as a negative control. The protein expression level was evaluated both the staining intensity and percentage of positive cells. The percentage of positive cells was scored as follows: 0 (negative), <10% positive cells; 1 (weak), 11–30% positive cells; 2 (moderate), 31–70% positive cells, and 3 (strong), >70% positive cells. The staining intensity was stratified as follows: 0, negative (no staining); 1, weakly positive (pale yellow staining); 2, moderately positive (yellow staining); and 3, strongly positive (brown-yellow staining). The sum of the staining intensity and positive cell scores was defined as the final score (staining index) for each section. The average STAT3 and FGFR2 staining index between TNBC and non-TNBC was compared.
