# Supplementary material for: FGFR2/STAT3 Signaling Pathway Involves in the Development of MMTV-Related Spontaneous Breast Cancer in TA2 Mice
Source: Front Oncol. 2020 May 5;10:652. doi: 10.3389/fonc.2020.00652 (PMC7214838; doi:10.3389/fonc.2020.00652)
Supplement: Supplementary file 2 [file Data_Sheet_2.doc]

**Supplementary table 1. Sequences of small interfering RNAs (siRNA) targeted to the STAT3.**

| **siRNA** | **Sense (5’-3’)** | **Antisense (5’-3’)** |
| --- | --- | --- |
| STAT3i-2315 | GAGGAGGCAUUUGGAAAGUTT | ACUUUCCAAAUGCCUCCUCTT |
| STAT3i-1415 | GGGUCUCGGAAAUUUAACATT | UGUUAAAUUUCCGAGACCCTT |
| STAT3i-1107 | CCCGCCAACAAAUUAAGAATT | UUCUUAAUUUGUUGGCGGGTT |
| STAT3i -PC | CACUCAAGAUUGUCAGCAATT | UUGCUGACAAUCUUGAGUGAG |
| STAT3i -NC | UUCUCCGAACGUGUCACGUTT | ACGUGACACGUUCGGAGAATT |

PC: GAPDH positive control; NC: Negative control, STAT3i: STAT3 knockdown.

**Supplementary table 2.** Detail information of antibodies used in western blot (WB), immunocytochemical (ICC) staining and immunohistochemical (IHC) staining.

| Antibodies | Companys | Dilution | | |
| --- | --- | --- | --- | --- |
|  |  | WB | ICC | IHC |
| FGFR2 | Proteintech | 1:1000 | * | 1:200 |
| STAT3 | Proteintech | 1:1000 | 1:600 | 1:300 |
| p-STAT3^Ser727^ | Bioss | 1:500 | 1:300 | 1:300 |
| HER-2 | MXB Biotechnologies Inc. |  |  | ready-to-use |
| PR | MXB Biotechnologies Inc. |  |  | ready-to-use |
| ER | MXB Biotechnologies Inc. |  |  | ready-to-use |
| p-STAT3^Try705^ | Bioss | 1:1000 | 1:400 | * |
| Bcl2 | Santa | 1:1000 | 1:500 | * |
| CyclinD1 | Bioss | 1:1000 | 1:1000 | * |
| c-myc | Santa | 1:500 | 1:100 | * |
| GAPDH | Proteintech | 1:3000 | * | * |
